# Supplementary material for: Bacteriophages from human skin infecting coagulase-negative Staphylococcus: diversity, novelty and host resistance
Source: Sci Rep. 2024 Apr 8;14:8245. doi: 10.1038/s41598-024-59065-9 (PMC11001980; doi:10.1038/s41598-024-59065-9)
Supplement: Supplementary file 1 — Supplementary Figures. [file 41598_2024_59065_MOESM1_ESM.pdf]

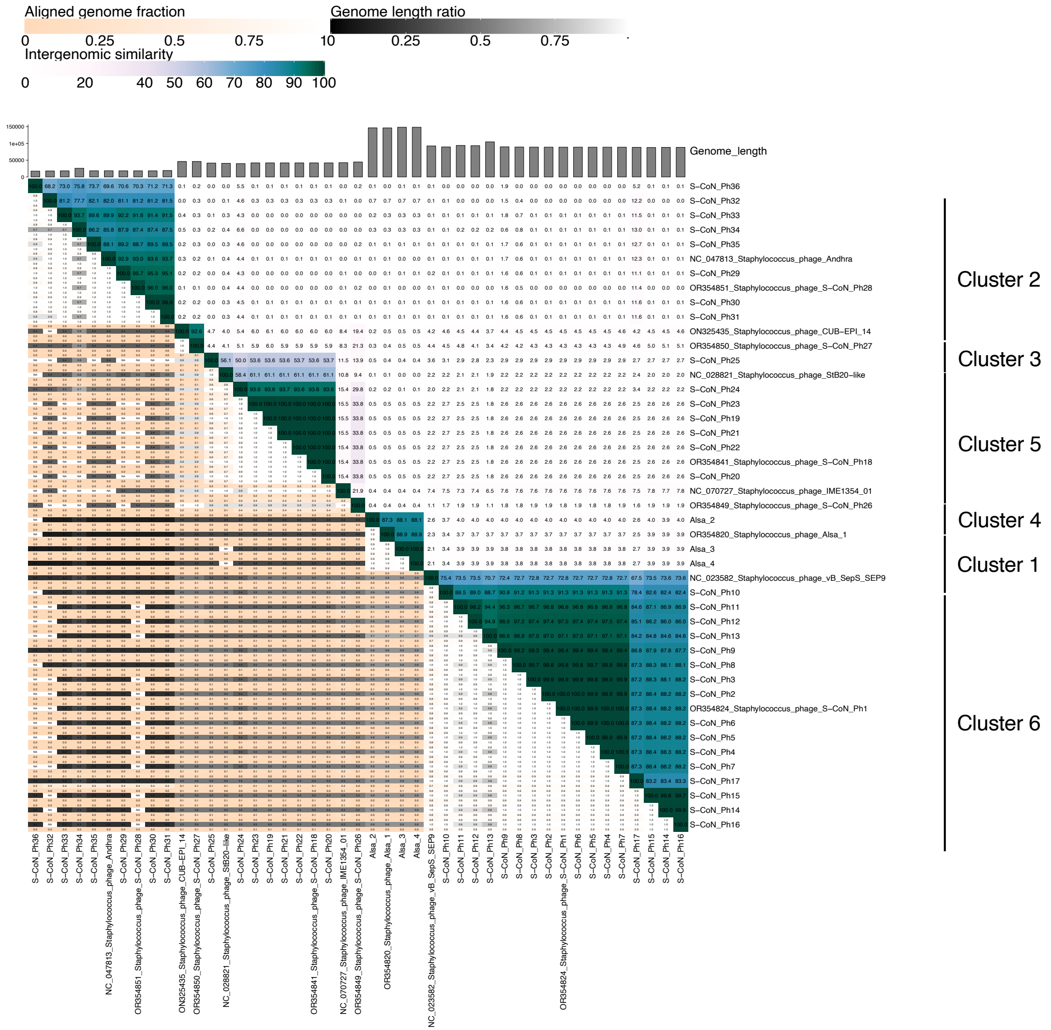

Figure S1: Intergenomic similarities analysis of CoNS phages

Heatmap generated using VIRIDIC to compare and cluster the 40 CoNS phages of this study. Intergenomic similarity between each pair of phage genomes is indicated on the right of the heatmap with values giving the similarity value (%) of each genome pair and blue-green shade gradient representing the degree of similarity. For each phage genome pair, three values are shown from top to bottom: aligned fraction of the first genome (in a row), the genome length ratio, and the aligned fraction of genome (column), respectively. Darker colour shows lower values indicating smaller aligned genome pairs (Orange to white colour range) or a large difference in the length of the genome pair (black to white scale). Published phage genomes were included as a reference for clusters 2-6 (cluster 2: NC_047813 ⌀Andhra; cluster 3: ON325435 ⌀CUB-EPI_14; cluster 4: NC_070727 ⌀IME1354_01; cluster 5: NC_028821 ⌀StB2—like; cluster 6: NC_023582 ⌀vB_SepS-SEP9). Staphylococcus phage IME1354_01 is a distinct species from ⌀ S-CoN_Ph26 (cluster 4) based on 95% definition of species.

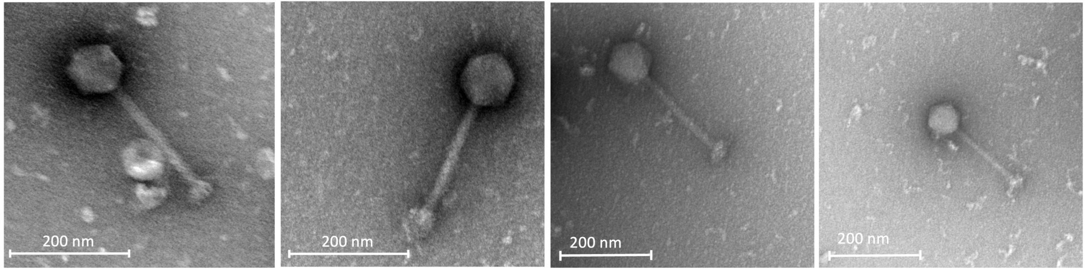

	Cluster	1			
	Phage	Alsa_1	Alsa_2	Alsa_3	Alsa_4
Mean ± SD	Tail length (nm)	200.59 ± 0.8	193.1 ± 0.6	215.26 ± 2.6	206.57 ± 1.3
	Head daim (nm)	88.96 ± 0.6	94.77 ± 0.7	98.10 ± 0.7	97.49 ± 0.4

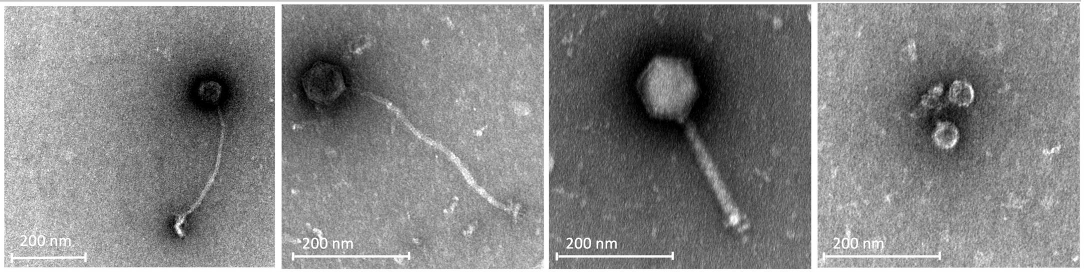

	Cluster	6		5	
	Phage	S-CoN_Ph12	S-CoN_Ph15	S-CoN_Ph21	S-CoN_Ph25
Mean ± SD	Tail length (nm)	330.980± 1.4	297.98 ± 2.5	178.24 ± 0.4	8.74 ± 1
	Head daim (nm)	77.36 ± 1.1	91.25 ± 2.2	98.92 ± 0.6	41.26 ± 0.5

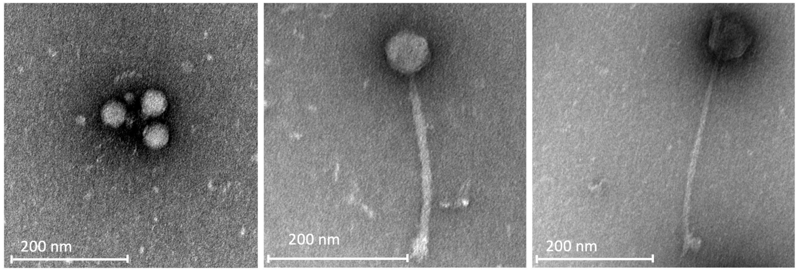

	Cluster	4	3	2
	Phage	S-CoN_Ph26	S-CoN_Ph27	S-CoN_Ph35
Mean ± SD	Tail length (nm)	7.35 ± 0.4	223.09 ± 1.4	303.95 ± 1.3
	Head daim (nm)	41.19 ± 0.4	59.39 ± 0.3	79.72 ± 1.4

Figure S2: Electron micrographs of Staphylococcus phages. Representative images of phages from the 6 clusters are shown, and head and tail measurements of phages were determined using imageJ from a minimum of three independent measurement.

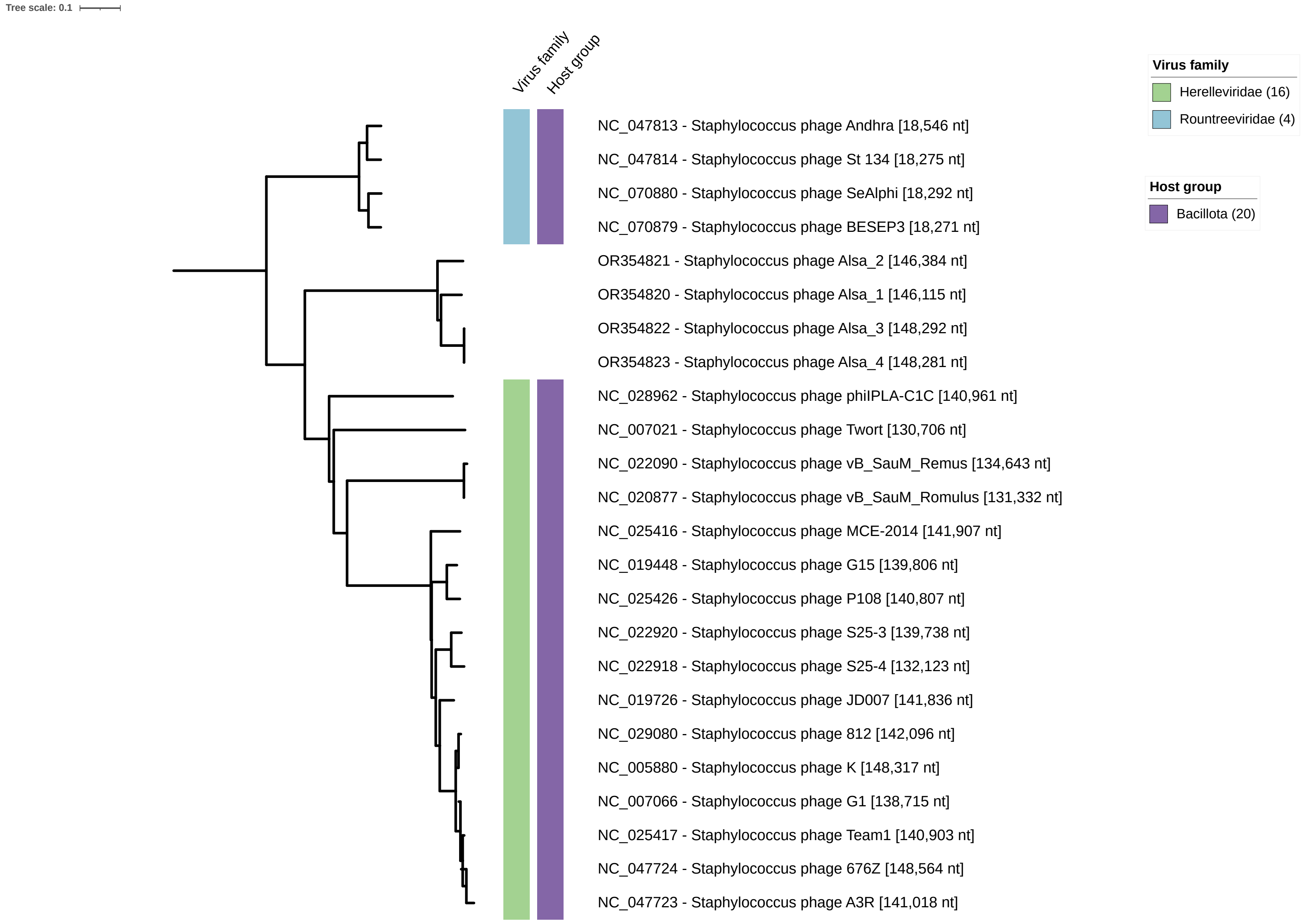

Figure S3: Phylogenic tree of Alsa and closely related *Staphylococcus* phages. Genomes of Alsa phages belong to the *Herelleviridae* virus family based on the tree generated by VIPTree 3.1 including closely related virus taxa calculated from genome distance matrix. Virus family taxonomy is indicated in blue (*Herelleviridae* infecting *S. aureus*) and green (other families) of the phylum Bacillota (host group).

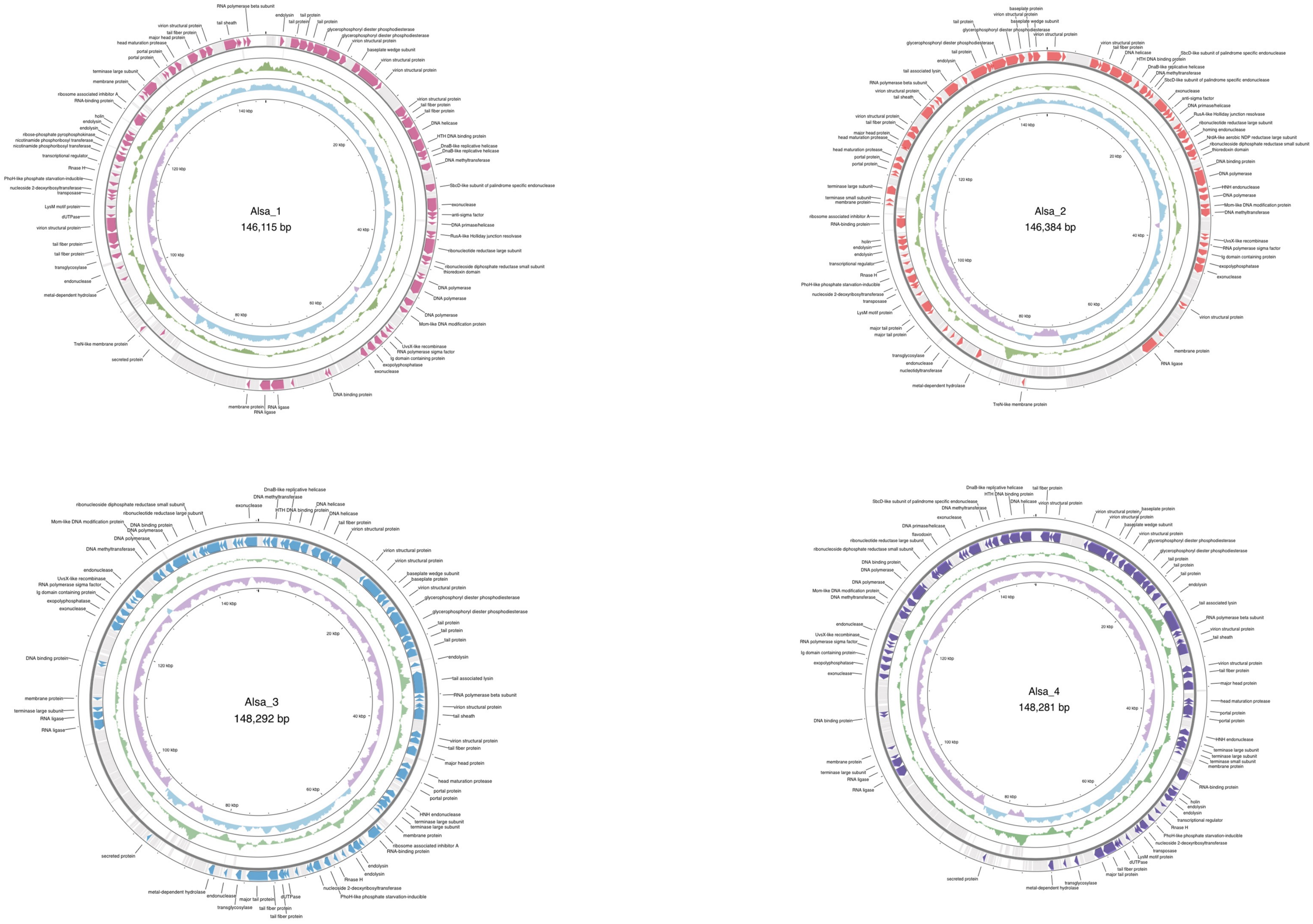

Figure S4: Circular maps of Alsa phage genomes. Predicted ORFs and annotation was performed using Pharokka. Phage genome ORFs are coloured pink (øAlsa_1), red (øAlsa_2), blue (øAlsa_3) and purple (øAlsa_4), with hypothetical proteins labeled grey. Inner rings show GC content (green), GC skew + (blue), and GC skew – (light purple).

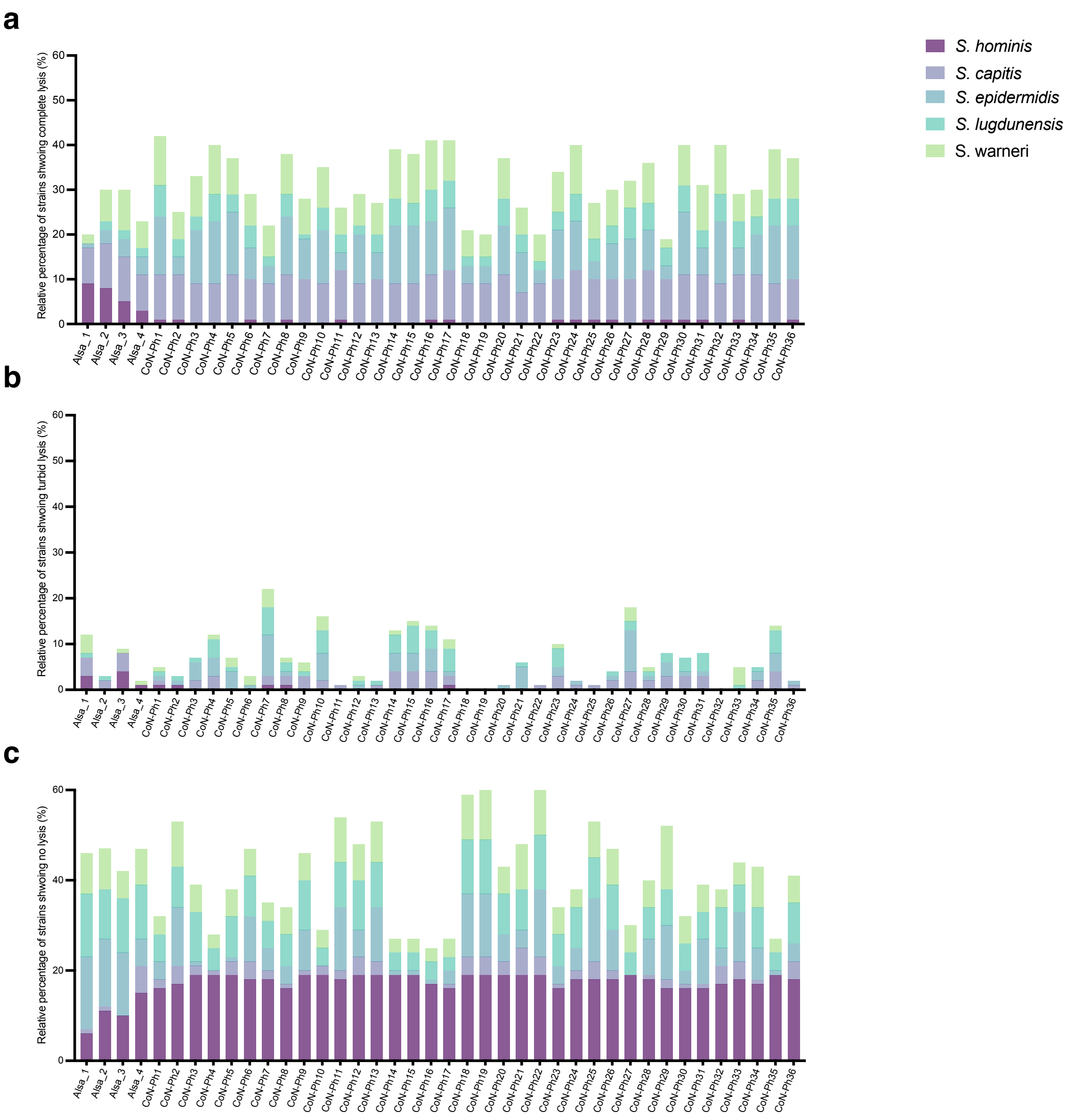

Figure S5: Comparison of host species lysis. From the host range assay spot assays, the relative percentage of each host strain with complete lysis zones after phage infection is shown for each of the 40 CoNS phages. CoNS hosts are indicated by the colours shown in the key.

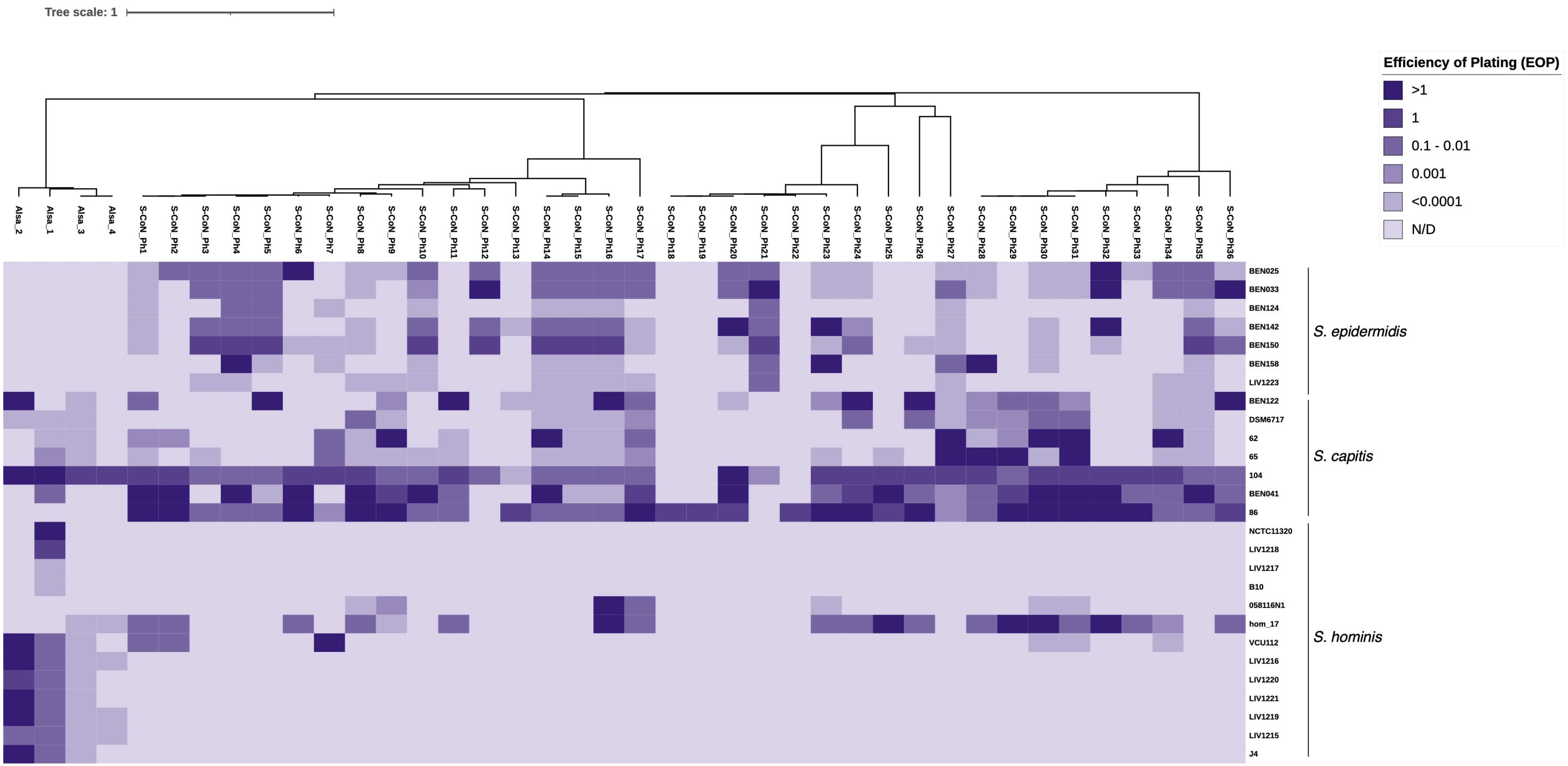

Figure S6: EOP assays of phages using hosts *S. epidermidis*, *S. capitis* and *S. hominis*. Representative strains of each host that showed complete lysis in the spot assay were tested for their ability to produce plaques, as calculated by PFU. EOP values were calculated relative to PFU values from propagation in their original hosts. This assay was repeated independently three times.

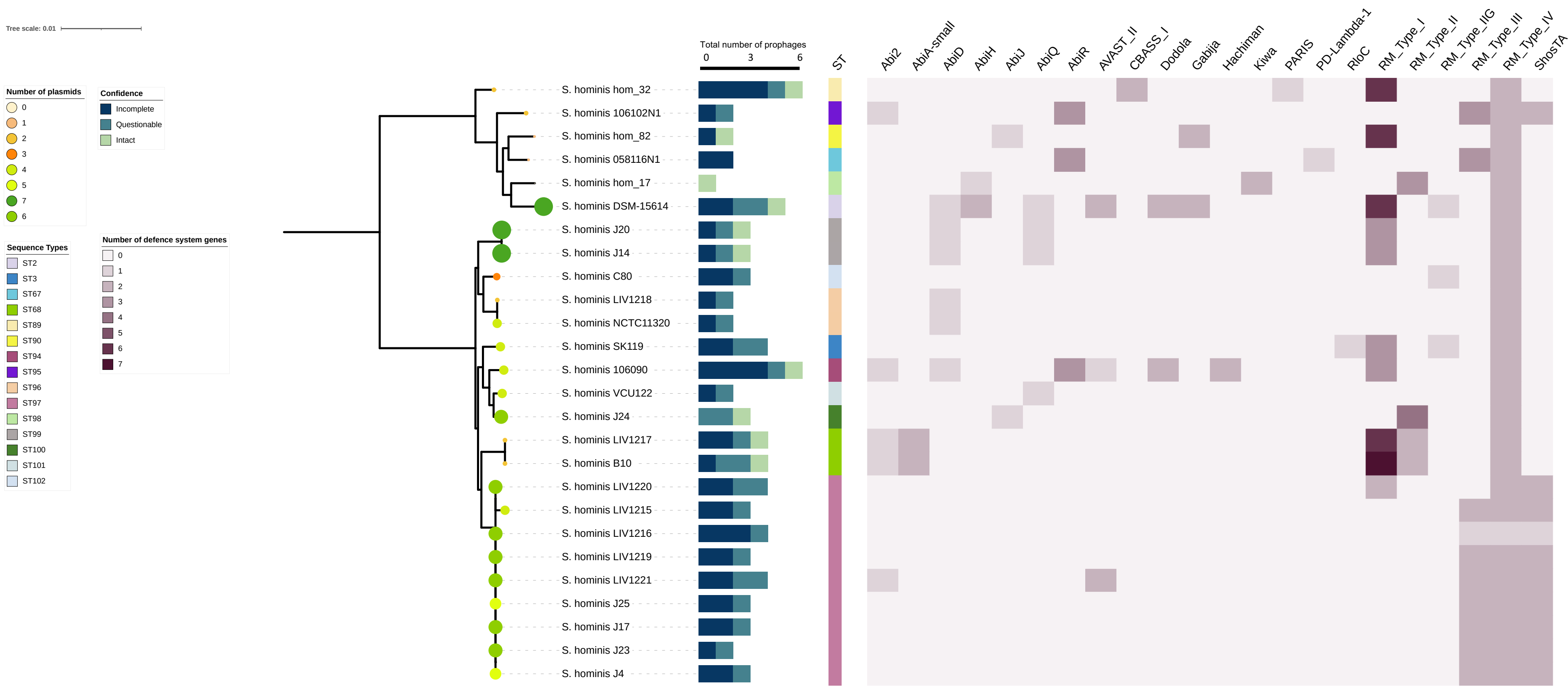

Figure S7: Phage defence systems and plasmid content of *S. hominis.* Phage defence system genes in genomes of the *S. hominis* strains were detected using DefenseFinder. Number of defence system genes present is scored (0-2 max from the key) and the total number of prophages (0-6) are indicated as either intact, incomplete, or questionable. Phylogenetic tree of *S. hominis* strains was generated using IQ-TREE. Sequence type (ST) of strains is indicated by vertical-coloured bars. Plasmid content (0-7 plasmids) is shown by different sized and coloured circle symbols, determined by PlasmidFinder.

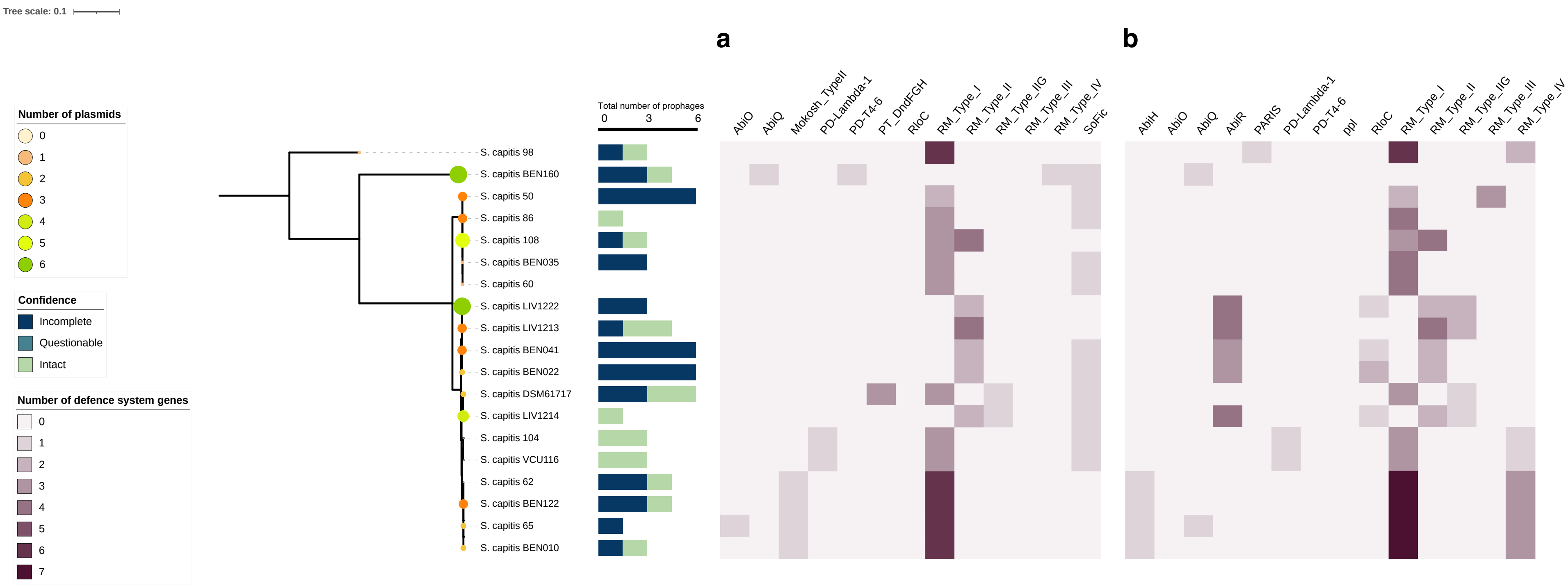

Figure S8: Phage defence systems and plasmid content *S. capitis.* Phage defence system genes present in the genomes of *S. capitis* strains were detected using (a) PADLOC and (b) DefenseFinder. Number of defence system genes present is scored (0-6), and the total number of prophages (0-6) are indicated as either intact, incomplete, or questionable. Phylogenetic tree of *S. capitis* strains was generated using IQ-TREE. Plasmid content (0-6 plasmids) is shown by different sized and coloured circle symbols, determined by PlasmidFinder

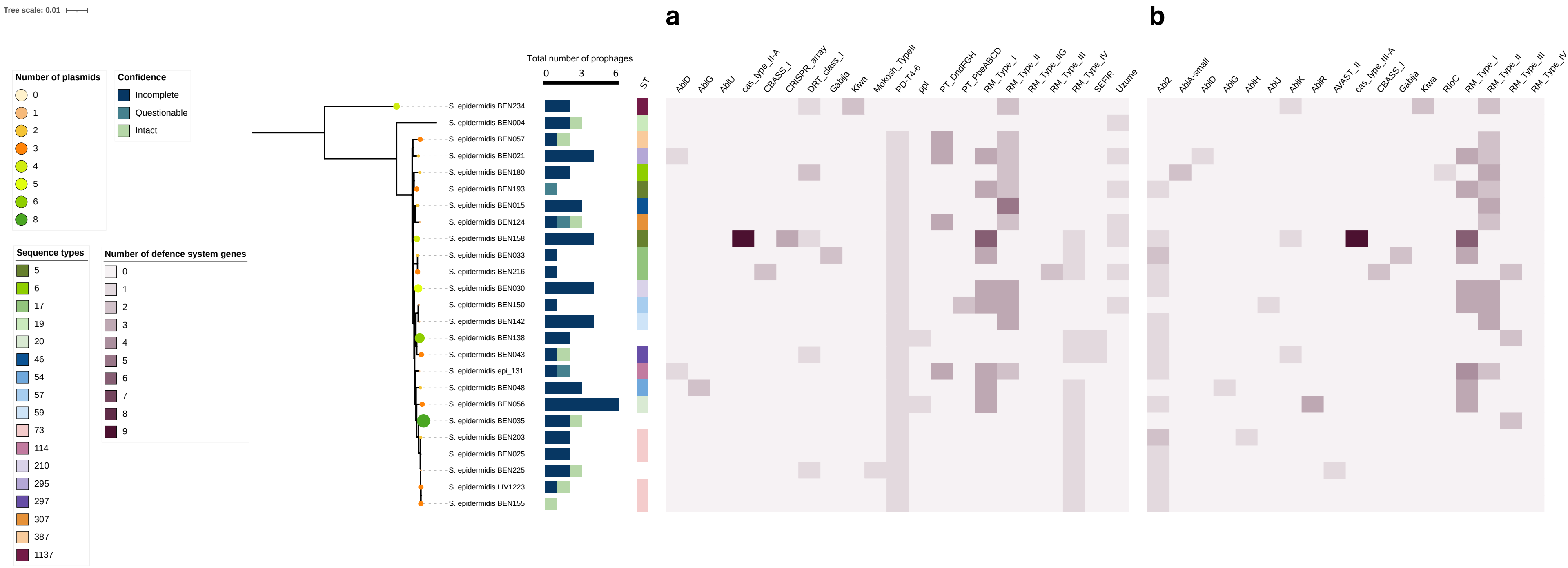

Figure S9: Phage defence systems and plasmid content *S. epidermidis*. Phage defence system genes present in the genomes of *S. epidermidis* strains were detected using (a) PADLOC and (b) DefenseFinder. Number of defence system genes present is scored (0-9), and the total number of prophages (0-6) are indicated as either intact, incomplete, or questionable. Phylogenetic tree of *S. epidermidis* strains was generated using IQ-TREE. Sequence type (ST) of strains is indicated by vertical-coloured bars. Plasmid content (0-8 plasmids) is shown by different sized and coloured circle symbols, determined by PlasmidFinder

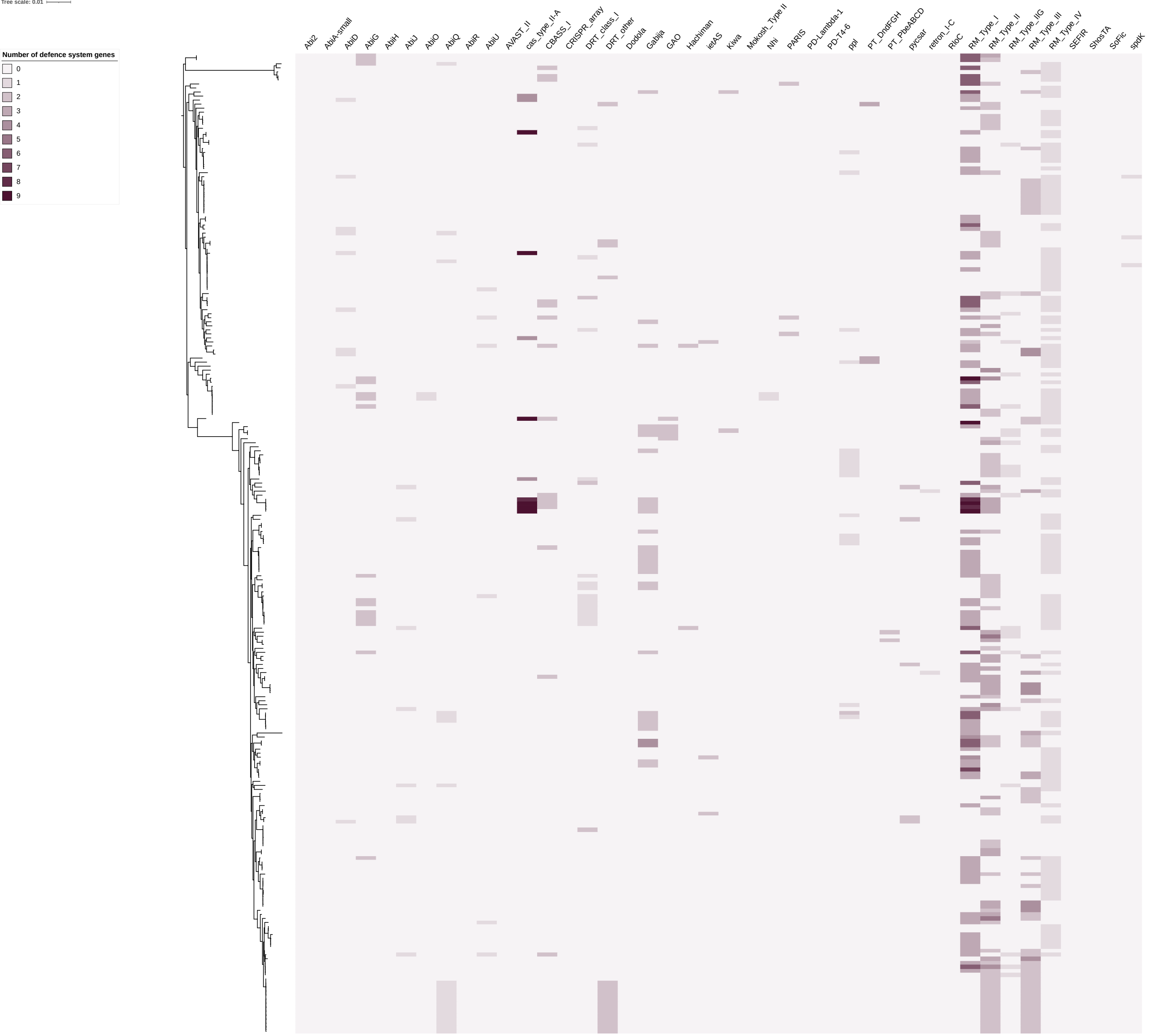

Figure S10: Antiviral defence systems of publicly available *S. hominis* genomes. Phage defence system genes of 243 *S. hominis* strain genomes from NCBI database were determined using PADLOC. Number of defence system genes present is scored (0-9).

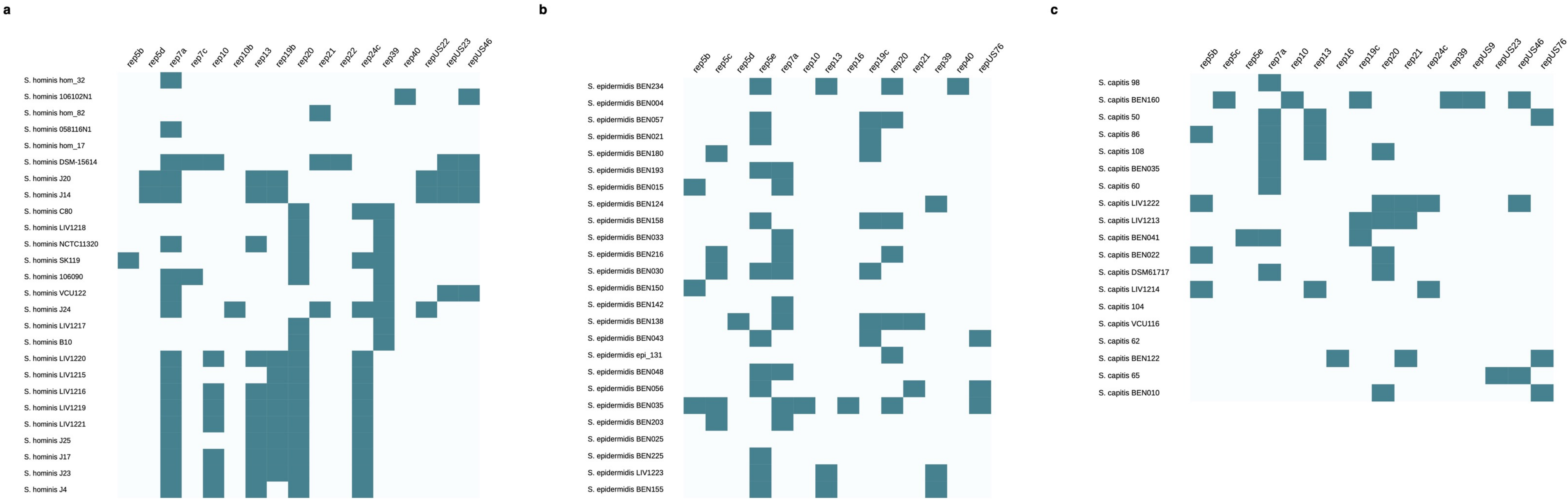

Figure S11: Presence of plasmid replicons in *Staphylococcus* species. Plasmids replicons present (dark blue) or absent in *S. hominis* (a), *S. epidermidis* (b), and *S. capitis* (c) strains used in the host range analysis were detected using Plasmid Finder.
